# Determining the feasibility of a trial to evaluate the effectiveness of phototherapy versus placebo at reducing pain during physical activity for people with knee osteoarthritis: a pilot randomized controlled trial 

**DOI:** 10.1186/s40814-020-00729-4

**Published:** 2020-11-26

**Authors:** Kyle Vader, Abey Bekele Abebe, Mulugeta Bayisa Chala, Kevin Varette, Jordan Miller

**Affiliations:** 1 School of Rehabilitation Therapy, Queen’s University, Kingston, ON Canada; 2 Chronic Pain Clinic, Kingston Health Sciences Centre, Kingston, ON Canada

**Keywords:** Phototherapy, Knee osteoarthritis, Sensitivity to physical activity, Pain, disability

## Abstract

**Background:**

Although practice guidelines recommend physical activity and exercise for the management of knee osteoarthritis, pain is a common barrier to participation. Phototherapy has been shown to reduce pain intensity for people with knee osteoarthritis, but it is unclear if it reduces pain during physical activity or contributes to improved rehabilitation outcomes.

**Objective:**

The aim of this study is to assess the feasibility of performing a fully powered randomized controlled trial (RCT) comparing an active phototherapy intervention versus placebo on pain during physical activity for people with knee osteoarthritis.

**Methods:**

A pilot RCT was conducted to test the feasibility of a trial comparing 8-sessions (4 weeks) of active phototherapy versus placebo. People were able to participate if they (1) were an English speaking adult (> 18 years of age), (2) had received a diagnosis of knee osteoarthritis from a physician, and (3) self-reported experiencing pain and disability related to their knee osteoarthritis for > 3 months. Primary outcomes were the feasibility of participant recruitment, retention, assessment procedures, and maintaining high treatment fidelity. Secondary outcomes piloted for a full trial included pain during physical activity (primary outcome of full trial); self-reported pain severity, physical function, stiffness, adherence to prescribed exercise, global rating of change, patient satisfaction, and adverse events; 6-min walk test; and pressure pain threshold.

**Results:**

Twenty participants (4 men; 16 women) with knee osteoarthritis and a mean age of 63.95 (SD: 9.27) years were recruited over a 3-week period (6.7 participants per week). Fifteen out of 20 (75%) of participants completed the primary outcome assessment at 4 weeks and 19/20 (95%) of participants were retained and completed the final 16-week assessment. Overall, 89% of all assessment items were completed by participants across all time-points. Fifteen out of 20 participants (75%) completed all 8 treatment sessions. Treatment fidelity was 100% for all completed treatment sessions. No adverse events were reported by participants in either group.

**Conclusions:**

Results suggest that the trial methodology and intervention are feasible for implementation in a fully powered randomized controlled trial to determine the effectiveness of phototherapy at reducing pain during physical activity for people with knee osteoarthritis.

**Trial registration:**

ClinicalTrials.gov, NCT04234685, January 21, 2020–Retrospectively registered.

## Key messages regarding feasibility

- Prior to this research, the feasibility of performing a fully powered randomized controlled trial comparing an active phototherapy intervention versus placebo to determine the effectiveness of phototherapy at reducing pain during physical activity for people with knee osteoarthritis was unknown.
- Results suggest the trial methodology and intervention are feasible for implementation in a fully powered randomized controlled trial to determine the effectiveness of phototherapy at reducing pain during physical activity for people with knee osteoarthritis.
- Results have the following implications for a fully powered randomized controlled trial: (1) we will aim to recruit participants from primary care sites where they have the physical space available to complete the 6-min walk test onsite when necessary to overcome geographic barriers to in-person assessments in order to achieve higher rates of completion for the primary outcome; (2) we will follow-up multiple times via email and telephone with participants who are unable to attend in-person assessments to ensure that we still achieve high rates of completion of self-report measures; and (3) we will update our sample size calculation in order to allow for up to 25% dropout from in-person sessions and/or assessments.

## Introduction

Musculoskeletal disorders are the leading contributor to years lived with disability worldwide [1–3]. Musculoskeletal disorders also have a significant economic burden on health care systems [4] and an even greater impact on society due to time off work [5–8]. As the number of older adults grows, the burden of osteoarthritis (OA) is only projected to rise [4].

Clinical practice guidelines recommend aerobic and strengthening exercise for people with knee OA [9–15]. Exercise is an effective non-surgical intervention for OA that can decrease pain and improve physical function [9–15]. However, adherence to physical activity and exercise interventions are low in this population [16–19]. Pain during physical activity is a frequently cited barrier that can interfere with adherence [20–22]. Effective treatment options to reduce pain during physical activity may help overcome barriers to participation in physical activity and exercise among this population.

Evidence suggests that pain with movement or physical activity may be a distinct construct from pain at rest [23] including differing mechanisms and pain processing [24–26]. There is currently a dearth of evidence on the effect of clinical interventions on reducing pain during physical activity; however, some early evidence suggests that pain during physical activity may respond differently to interventions than pain at rest. For example, evidence suggests that opioids, commonly used in the treatment of post-surgical pain, have a very small impact on pain during movement for people with post-surgical pain [27]. On the other hand, transcutaneous electrical nerve stimulation (TENS) [28] and acetaminophen [29] have been demonstrated to be potential modulators of pain during movement. Pain during physical activity is often not assessed as a distinct construct in clinical trials on OA, but is an important barrier to participation in physical activity and exercise interventions that requires further study.

Phototherapy may help to address the need for effective, non-pharmacological, pain-relieving interventions in people with knee OA [30–32]. Although the physiological mechanisms of pain relief are not clear, phototherapy has been shown to reduce inflammation, induce analgesia, and promote healing of musculoskeletal disorders [33, 34]. One hypothesis is that phototherapy can increase nociceptive thresholds, which can result in subsequent pain relief [35]. Phototherapy has demonstrated improvements in pain for several musculoskeletal disorders such as low back and neck pain [36–40], suggesting it may be a valuable component in the management of knee OA. However, research on the effectiveness of phototherapy on pain during physical activity remains unknown. Before proceeding with a fully powered randomized controlled trial to evaluate the effectiveness of phototherapy on pain during physical activity, we set out to determine the feasibility of conducting such a trial.

Primary outcomes of this pilot study were feasibility measures including participant recruitment rate, assessment completion rate, retention rate, and treatment fidelity. Secondary outcomes piloted for a full trial included pain during physical activity (primary outcome for the full trial), pain severity, pressure pain threshold, functional walking performance, and self-reported physical function stiffness, pain, adherence to prescribed exercise, global rating of change, patient satisfaction, and adverse events.

## Methods

### Design

The study was designed as a parallel group, pilot randomized controlled trial with 1:1 allocation ratio to active phototherapy intervention or placebo.

### Recruitment

Participants were recruited from two primary care practices in Kingston, Ontario, Canada. People who called the primary care clinics were asked for their “reason for visit” (as is standard practice). When the reason for visit was knee OA, people were asked by their physician or nurse if they were willing to be contacted by a research assistant to be invited to participate in this research. If they agreed to being invited to participate, they were asked to provide contact information for a research assistant to contact them. The research assistant then called them, screened them for eligibility over the telephone, and invited eligible participants to an initial visit where agreeable participants provided informed consent and completed an initial assessment.

### Sample size

Our targeted sample size was 20 participants. As this was a pilot study, a formal sample size calculation was not conducted. A sample of 20 participants was chosen because researchers and clinical partners felt this would be a large enough sample to allow an accurate assessment of the feasibility of recruitment, retention, outcome measure completion, and delivering the phototherapy and exercise interventions. Additionally, this sample size meets the recommendation from Whitehead et al. of a minimum of 10 participants per arm when estimating the variance for a sample size calculation for a future trial designed with 90% power, two-sided 5% significance, and a large (0.8) effect size [41]. Estimating the variance was not a primary objective of this study and given the uncertainty of the variance estimated from such a small sample, variance estimates from previous research on interventions to improve pain and function for people with knee OA will also be used to inform our sample size for the future trial.

### Inclusion criteria

English speaking adults (> 18 years of age) who had been given a diagnosis of knee OA by a physician and had experienced pain and disability related to their knee OA for at least 3 months in duration were able to participate in this research.

### Exclusion criteria

Participants were excluded if they had a skin condition or other condition for which the application of phototherapy may have been contra-indicated (e.g., skin cancer or people receiving radiation therapy), history of total joint arthroplasty in the affected knee, and “red flags” suggestive of non-musculoskeletal etiology (i.e., fever/chills, unrelenting night pain, multi-segmental or bilateral loss of sensation, or sudden and unexplained weight loss).

### Research ethics

This study was approved by the Queen’s University Health Sciences and Affiliated Teaching Hospitals Research Ethics Board in Kingston, Ontario, Canada (Reference Number: 6022509). All participants provided written informed consent prior to participation in this research.

### Randomization and allocation concealment

Participants were randomized using a computer-generated block random number generator with random block sizes by a study team member not involved in the assessments, allocation, or interventions. The allocation sequence was recorded on cards and placed in sequentially numbered opaque envelopes that were sealed. Allocation was concealed until after the initial assessment was completed. A research assistant opened the sealed envelope that said either “group A” or “group B” after the initial assessment without knowledge of whether “group A” or “group B” would receive the active or placebo phototherapy.

### Blinding

Participants and research assistants were blinded to whether participants received the active phototherapy intervention or placebo. The phototherapy machine was pre-programmed with two dosage settings for “group A” or “group B.” Both settings involved emission of a visible red light. The phototherapy started after a programmed 15-s delay to allow the research assistant to exit the room prior to the treatment starting. This prevented the research assistant from seeing the difference in light intensity between intervention and control groups. Whether the “group A” or “group B” was the active phototherapy or placebo dosage was not revealed to the research assistant or participant at any point during the research study.

### Intervention

#### Exercise and education

Participants from both the active phototherapy intervention and placebo groups took part in a standardized education and exercise intervention as suggested by best evidence and clinical practice guidelines for knee OA [12, 15]. These sessions were approximately 20 min in length and took place twice weekly for 4 weeks (8 sessions) with a trained physiotherapist.

##### Education

Participants received education on evidence-based management of knee OA at the first visit [12, 15]. This education included describing the importance of regular physical activity in managing knee OA and how our bodies can adapt to gradual increases in activity and exercise. It also included education on how temporary increases in pain or soreness after activity can be normal when starting an exercise program. The education was provided at the first treatment session and took approximately 10 min.

##### Aerobic exercise

Participants were provided with a tailored home walking program [10, 42]. Participants were given instructions at their initial visit and these instructions were re-inforced at each subsequent visit. For those who were able, participants were instructed to walk 30 min 5 times per week in order to meet physical activity guideline recommendations [43, 44]. All participants who reported being unable to achieve this walking duration (e.g., pain or physical disability interfered with the ability to walk for 30 min) were provided with a progressive walking program [45]. The progressive walking program started at their baseline, defined as the distance they felt confident they could walk without being sore an hour later and without feeling like they would be unable to perform their usual activities of daily living during the remainder of the day because of the walk. Participants worked with the physiotherapist to develop a progressive walking program that progressed by 10–20% each week from this baseline until they reached 30 min per day.

##### Strengthening, balance, and range of motion exercises

Participants were instructed how to perform a series of six land-based exercises that were consistent with clinical practice guidelines for knee OA [11, 12, 15], including strengthening (squat to chair, step-up, lunge, resisted knee flexion), balance (single-leg stance), and range of motion (terminal knee flexion/extension range of motion) exercises. The exercise technique and dosage were tailored to the participant’s current abilities under the supervision of a physiotherapist. For example, if a participant was unable to squat down to the level of a chair and return to standing, the chair height was raised until the participant was able to perform the activity at least 8 times. The single-leg balance was modified to a tandem stance if a participant was unable to perform a single leg stance. The participants were asked to complete two to three sets of 8–12 repetitions of each exercise, once daily. Participants performed the exercises twice/week in the lab under the supervision of a physiotherapist where they received support for exercise progression and problem solving any difficulties encountered with the exercises.

#### Active phototherapy group

The active phototherapy intervention group received phototherapy in addition to the education and exercise intervention twice weekly for 4 weeks. Participants were positioned in a comfortable and relaxed position on a therapy plinth. The *invitalizer 3.0* workstation was used to apply High Intensity Physio Light (HIPL™) therapy (*invitalize*, Kitchener, Ontario, Canada). The phototherapy illuminated the affected knee for 20 min. In the case of bilateral knee OA, the treatment was applied to both knees (20 min each). The knee was positioned approximately 2.54–7.62 cm from the phototherapy source with an intensity setting of 50 mW/cm^2^. The *invitalizer 3.0* workstation uses 5 panels of lights with a total emitter array area of 800 cm^2^. The 5 panels can be easily configured to allow the light source to be positioned consistently 2.54–7.62 cm from the skin over the anterior, medial, and lateral sides of the knee during application regardless of the size or shape of the participant’s knee.

#### Placebo group

The control group received the same procedure as the active phototherapy intervention group (e.g., education and exercise), with the substitution of 20 min of placebo phototherapy (versus active phototherapy intervention) twice weekly for 4 weeks. The intensity of the HIPL*™* therapy was set at 1 mW/cm^2^ for the placebo group using the *invitalizer 3.0* workstation (a dosage at which there was no therapeutic benefit expected, but the light was still be visible to the participant).

### Assessment and outcomes

The feasibility outcomes described below were the primary outcomes of this pilot study. Secondary outcomes included the outcomes planned for the full trial. We determined the feasibility for collecting these outcomes and report them descriptively with no statistical comparisons made between groups.

#### Feasibility outcomes

##### Recruitment

The feasibility of participant recruitment was determined by the overall recruitment rate. A full trial was considered feasible with our current recruitment methods if, during the pilot study, we were able to recruit three participants per week. This recruitment rate would allow us to exceed the estimated 168 participants needed for a fully powered trial in a 56-week recruitment period.

##### Retention

Retention was assessed by attrition rate, with < 20% attrition at 3-month follow-up considered indicative of feasibility with our current protocol. We decided a priori that a full trial could be conducted with attrition rates of < 30%, but with additional strategies to increase retention. This is based on evidence that > 20% attrition threatens trial validity [46].

##### Assessment procedures

Feasibility of the assessment procedures were measured by completeness of data and duration of completing all outcome measures. As recommended for pilot studies, we set criteria a priori for acceptable completeness [47] and considered > 80% of all assessment items completed and a mean time for completion of < 60 min as acceptable.

##### Treatment fidelity

Treatment fidelity was encouraged through training the physiotherapists delivering the program and by providing a treatment fidelity checklist to the research assistants/physiotherapists. Fidelity was measured through an audit of the fidelity checklist and reported as a proportion of intervention components (education, exercise, phototherapy) delivered in alignment with the protocol. An a-priori acceptable level of fidelity was considered 80% for each component of the intervention (education, exercise, and phototherapy) among participants that attend each visit.

#### Participant characteristics

The following measures were collected at baseline to describe the study population: age, sex, gender, duration of knee pain, current medications, comorbidities (using a standardized disease count to measure comorbidity [48, 49]), work status, annual household income, and number of other painful body sites.

#### Primary outcome piloted for the full trial

##### Pain during physical activity

Pain during physical activity was measured using change in pain throughout a standardized physical activity, a measure called sensitivity to physical activity (SPA) in the literature [50–53]. SPA has been shown to explain unique variance in self-reported disability beyond that explained by static pain measure s[54], suggesting that there may be clinical value to measuring SPA as a distinct construct from pain at rest. In this study, SPA was measured using change in pain throughout a standardized 6-min walk test (6MWT) [50, 53]. Pain intensity was measured each minute throughout the 6MWT. The SPA index was calculated as the difference between the peak pain intensity during the 6MWT and baseline pain intensity at rest immediately prior to the test. SPA will be the primary outcome for the full trial.

#### Secondary outcomes piloted for the full trial

##### Pain severity

Pain severity was measured using a Numeric Pain Rating Scale (NPRS) with anchors including no pain (0) to worst imaginable pain (10). Participants were asked to report their pain intensity at rest currently, on average over the last 24 h, at its worst over the last 24 h, and at its least over the last 24 h. The pain severity was calculated as the mean of the four items. This combination of questions has been validated as a symptom severity index in the Brief Pain Inventory [55].

##### WOMAC physical function subscale

Self-reported physical function was measured by the Western Ontario and McMaster Universities Osteoarthritis Index (WOMAC) [34, 56] physical function subscale (0 to 68) with higher scores indicating greater functional limitations. The WOMAC physical function subscale uses a series of questions about ability to perform functional tasks answered on a four-point Likert scale.

##### WOMAC pain subscale

The WOMAC pain subscale (0–20) was collected as a secondary pain intensity measure with higher scores indicating greater pain [56]. The WOMAC pain subscale asks participants to report their pain intensity during walking, stairs, in bed, sitting or lying, and standing on a four-point Likert scale. The WOMAC was chosen as the most appropriate physical function measure, but the WOMAC also includes pain and stiffness subscales. The pain subscale will be considered secondary to the pain severity index as a pain measure.

##### WOMAC stiffness subscale

Stiffness was reported by participants on the WOMAC stiffness subscale (0–8) with higher scores indicating greater reports of stiffness [56]. The WOMAC stiffness subscale is a two-item scale asking participants to report their stiffness after waking up and later in the day on a four-item Likert Scale.

##### Adherence to prescribed exercise

Adherence to prescribed exercise was measured using a self-reported daily log of all exercises prescribed throughout the 4-week treatment period. The log included whether each prescribed exercise was performed, the number of repetitions, and the number of sets of each exercise. The outcome is reported as proportion of prescribed exercises completed for each participant.

##### Global rating of change

Global rating of change (GROC) was measured using an 11-point global rating of change scale (GROC) (− 5 to + 5) as has been recommended in the literature for self-reported rating of change [57, 58].

##### Patient satisfaction

Satisfaction was assessed using an 11-point scale with anchors of very dissatisfied (− 5) and very satisfied (+ 5).

##### Adverse events

Adverse events were recorded using an adverse events questionnaire that was reported consistently with reporting guidelines [59, 60] and asked (1) if the participant has experienced any adverse events as a result of any of the treatments received (yes/no); If any adverse events were reported, participants were asked the following: (2) what adverse events were experienced; (3) how long the event lasted (hours or days); and (4) how severe the adverse event was (0–10 scale).

##### Physical function performance

A performance measure of physical function was the 6-min walk test (6MWT) [61, 62]. The 6MWT is a test of walking speed, aerobic capacity, and endurance. It involves walking continuously on a 30-m walkway for 6 min. The score on the test was the distance walked. Distance walked was measured at the same time as the pain during physical activity measure.

##### Pressure pain threshold

Pressure was applied using a digital handheld pressure algometer with a 1 cm^2^ rubber tip (Wagner Instruments, Texas). Pressure was applied to eight test sites in the peripatellar region, and one control site 5 cm distal to the tibial tuberosity, as described by Ardent-Neielson and colleagues [63]. Site 1: 2 cm distal to the inferior medial edge of patella; site 2: 2 cm distal to the inferior lateral edge of patella; site 3: 3 cm lateral to the mid point on the lateral edge of patella; site 4: 2 cm proximal to the superior lateral edge of patella; site 5: 2 cm proximal to the superior edge of patella; site 6: 2 cm proximal to the superior medial edge of patella; site 7: 3 cm medial to the mid point on the medial edge of patella; and site 8: at centre of patella. At each site, participants were asked to report the moment the pressure switched from comfortable pressure to slightly unpleasant pain [64]. The pressure pain threshold was recorded in Newtons (N). The pressure pain threshold was calculated as the mean of measures across the 8 peripatellar sites and the pressure pain threshold at the standard tibialis anterior site was reported separately. Pressure pain threshold was piloted for the full trial in anticipation of including this as a process outcome to determine the relationships between pressure pain threshold (a measure of sensitivity to mechanical stimuli) and SPA.

### Timeline for assessment

Participant characteristics were collected at baseline. The primary outcome (SPA) was measured before and after phototherapy at baseline, 2 weeks (mid-treatment period), and 4 weeks (end of treatment period). Self-reported secondary outcomes (pain severity and WOMAC [physical function, pain, and stiffness subscales]) were assessed at baseline, 2, 4, 8, 12, and 16 weeks (baseline, mid-treatment, end of treatment, 4-, 8-, and 12-week follow-ups). Adherence was measured daily throughout the 4-week treatment period through a patient log with data collected at each visit. Global rating of change and satisfaction were measured at all time points after the baseline assessment. 6MWT was measured before and after treatment at baseline, 2 weeks (mid-treatment), and 4 weeks (end of treatment). Pressure pain threshold was measured prior to phototherapy at baseline and 4 weeks (end of treatment).

Current pain intensity and adverse events were also measured immediately prior to phototherapy, immediately after phototherapy (before exercises), and immediately following completion of exercise to determine the immediate effects of the phototherapy and the combined phototherapy and exercise intervention.

### Data collection and management

All baseline characteristics and outcomes were collected using Qualtrics online survey platform (Qualtrics, Provo, UT, USA, 2013). Assessments completed at baseline, 2 weeks, and 4 weeks were completed in-person with a blinded research assistant. Eight-, 12-, and 16-week assessments were completed through the online survey with individual links to the survey sent to participants and phone calls to remind participants to complete the surveys completed by the blinded research assistants. Multiple attempts were made to contact participants by phone or email for any participant who missed an assessment.

#### Analysis

As recommended for pilot studies [47], feasibility outcomes are reported in relation to pre-specified feasibility criteria and outcomes piloted for the full trial are reported descriptively in aggregate and by group without statistical comparisons between groups. Means and standard deviations are reported for normal, continuous data. Medians and interquartile range are reported for non-normal data. Counts and percentages are reported for categorical data.

## Results

Twenty adults with knee OA participated in this pilot study. The majority of participants were female (13/20) with a mean age of 63.95 (SD 9.27) years. Participant baseline characteristics are presented in Table 1.

**Table 1:** Baseline characteristics of study participants

Characteristics	Aggregated (*n* = 20)	Active phototherapy (*n* = 10)	Placebo control (*n* = 10)
Age–years (mean, SD)	63.95 (9.27)	60.60 (10.35)	67.30 (7.01)
Sex			
Male (*n*, %)	7(35)	3(30)	4(40)
Female (*n*, %)	13 (65)	7 (70)	6 (60)
Gender			
Man (*n*, %)	7 (35)	3 (30)	4 (40)
Woman (*n*, %)	13 (65)	7 (70)	6 (60)
Annual household income (*n*, %)			
< $20,000	0 (0)	0 (0)	0 (0)
$20,000 to $40,000	7 (35)	4 (40)	3 (30)
$40,000 to $60,000	3 15)	1 (10)	2 (20)
$60,000 to $80,000	2 (10)	1 (10)	1 (10)
$80,000 to $100,000	2 (10)	2 (20)	0 (0)
> $100,000	6 (30)	2 (20)	4 40)
Affected knee (*n*, %)			
Left	16 (80)	8 (80)	8 (80)
Right	4 (20)	2 (20)	2 (20)
Knee pain duration–years (mean, SD)	7.38(9.25)	7.15 (10.21)	7.60 (8.74)
Number of other body regions with pain (mean, SD)	4.00 (2.68)	3.90 2.64)	4.10 (2.85)
NSAID usage in the past 3 months			
Yes (*n*, %)	7 (44)	3 (42.90)	4 (44.44)
No (*n*, %)	9 (56)	4 (57.10)	5 (55.56)
Pain killer usage in the past three months			
Yes (*n*, %)	10 (63)	3 (42.90)	7 (77.78)
No (*n*, %)	6 (37)	4 (57.10)	2 (22.22)
Number of comorbidities (median, IQR)	0.80 (1)	0.70 (1)	0.90 (1)
Comorbidities (*n*,%)			
Heart attack	1 (5)	0 (0)	1 (10)
Asthma	3 15)	0 (0)	3 (30)
Chronic obstructive pulmonary disease	1(5)	0 (0)	1 (10)
Diabetes	7 35)	3 (30)	4 (40)
Kidney problems secondary to diabetes	1 (5)	1 (10)	0 (0)
Eye problems secondary to diabetes	1 (5)	1 (10)	0 (0)
Poor kidney function	1 (5)	1 (10)	0 (0)
Rheumatoid arthritis	2 (10)	1 (10)	1 (10)

*SD* standard deviation, *NSAID* non-steroidal anti-inflammatory drug, *IQR* interquartile range

### Feasibility outcomes

#### Recruitment rate

Twenty participants were recruited over 3 weeks (6.7 participants/week) from two primary care sites. Recruitment was stopped when the targeted sample of 20 participants was reached.

#### Retention

**Fig. 1:**
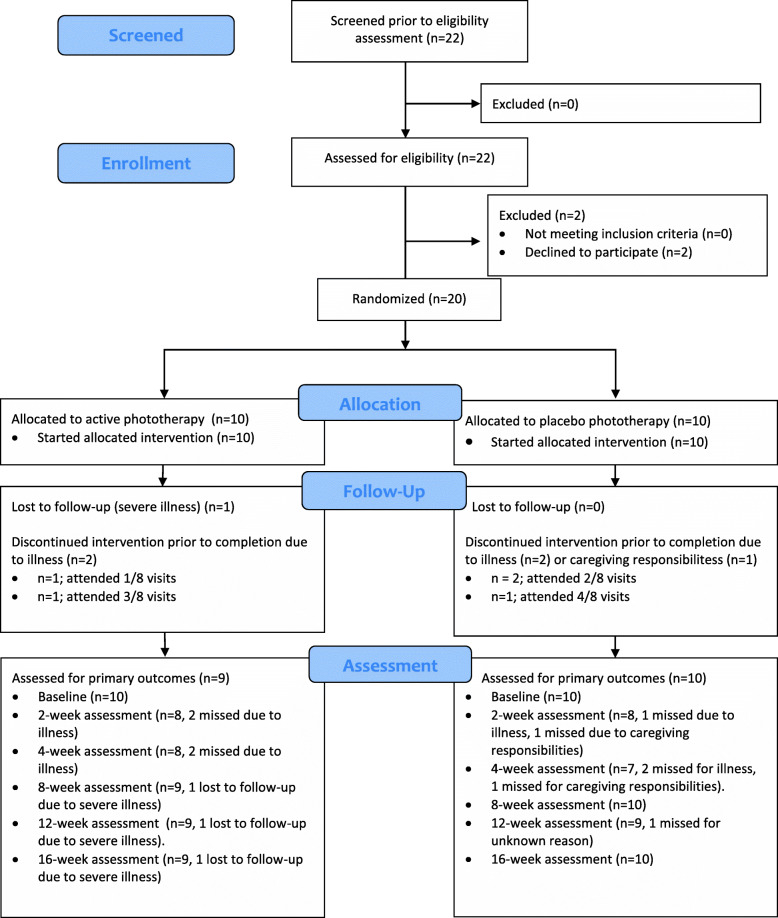
CONSORT flow diagram extension to randomized pilot and feasibility studies

We achieved an attrition rate of 5% at the end of the study with 19/20 participants completing the 12-week follow-up assessment at 16 weeks. The one person that did not complete the assessments at 16 weeks was from the active phototherapy group and was lost to follow-up due to severe illness. The CONSORT flow diagram extension for randomised pilot and feasibility trials is presented in Fig. 1 [60].

#### Assessment procedures

We achieved an 89% assessment completion rate across all time-points (89% of all assessment items completed). Of the completed assessments, we had an over 99% item completion rate with very few missing items. The completion rate was 100%, 80%, 75%, 95%, 90%, and 95% at the baseline, 2-, 4-, 8-, 12-, and 16-week assessments respectively. Importantly, the 2- and 4-week assessments relied on in-person appointments for completion of performance measures and psychophysical tests, whereas the 8-, 12-, and 16-week assessments were completed online or by phone. In addition to the participant who dropped out due to illness, the reasons reported for not completing the assessments were hospitalization (*n* = 1), caregiving responsibilities for a hospitalized spouse (*n* = 1), mental health concerns (*n* = 1), and surgical complications (*n* = 1). These concerns were reported to interfere with in person assessments, but all of these four participants completed the final assessment through online data collection or by phone. Of the 19 completed follow-up assessments at 16 weeks, 18 completed them using the unique link provided by email and one requested to complete the assessment by phone.

The mean time to completion for outcome measures was 14.4 min (SD 5.7) at baseline, 5.9 min (SD 3.4 min) at visit 4 assessment, and 5.7 min (SD 4.1) at visit 8 assessment. The mean time to completion at 8-week, 12-week, and 16-week follow-up was difficult to calculate due to delays evident within the survey (i.e., people leaving the survey open or leaving and coming back). Removing any surveys that took over 30 min as outliers, the following mean times to completion were calculated: 10.7 min, 14.25 min, and 21.2 min at 8-, 12-, and 16-week follow-ups respectively.

#### Treatment fidelity

Fifteen of 20 people completed all eight in-person visits (8/10 from the active phototherapy group and 7/10 from the placebo group). One person completed 4 visits, one person completed 3 visits, two people completed 2 visits, and one person completed 1 visit. The audit of the fidelity checklist suggested that the active phototherapy intervention and placebo groups were delivered with 100% consistency with the planned protocol for all visits in which participants attended. Similarly, all participants received education and exercise consistent with the protocol in both groups.

### Outcomes piloted for a full trial

We piloted the collection of primary and secondary outcomes for a full trial. Primary and secondary outcomes are reported in aggregate form in Table 2. The standard deviations from these results will help to inform sample size calculations for a full trial.

**Table 2:** Aggregated outcome measure data (from active phototherapy intervention and placebo control groups) at all assessment time-points

Measures	Baseline (week 0)			Visit 4 (week2)			Visit 8 (week 4)			4-week follow-up (week 8)			8-week follow-up (week 12)			12-week follow-up (week 16)		
	*N*	Mean	SD	*N*	Mean	SD	*N*	Mean	SD	*N*	Mean	SD	*N*	Mean	SD	*N*	Mean	SD
SPA (peak pain–baseline pain)																		
Pre	20	2.17	1.18	16	0.81	0.75	15	0.60	1.24	–	–	–	–	–	–	–	–	–
Post	20	1.33	1.15	16	0.56	0.81	15	0.53	1.13	–	–	–	–	–	–	–	–	–
Pain severity index																		
Worst	20	4.75	2.15	16	3.37	2.27	15	3.00	1.81	19	3.11	2.73	18	3.17	2.77	19	3.11	2.75
At least	20	1.70	1.63	16	0.69	1.07	15	0.20	0.41	19	1.37	2.45	18	1.39	2.42	19	1.16	2.36
Average	20	3.55	1.50	16	1.81	1.47	15	1.20	1.01	19	2.42	2.36	18	2.17	2.36	19	2.00	2.33
Right now	20	2.95	2.01	16	1.81	1.94	15	0.93	1.62	19	1.63	2.81	18	1.83	2.87	19	1.79	3.01
Index	20	3.24	1.61	16	1.92	1.45	15	1.33	0.90	19	2.13	2.53	18	2.14	2.52	19	2.01	2.48
WOMAC																		
Pain	20	6.30	2.34	16	4.31	2.44	15	3.60	2.35	19	4.58	3.77	18	5.00	4.11	19	4.53	4.03
Stiffness	20	3.10	1.70	16	2.25	1.53	15	1.67	1.11	19	2.89	1.62	18	2.67	1.97	19	1.79	1.75
Physical Function	20	22.95	9.72	16	15.81	10.52	15	9.913	6.81	19	17.32	16.66	18	17.83	16.99	19	13.63	14.79
Total	20	32.35	12.00	16	22.38	13.63	15	14.40	9.32	19	24.79	21.11	18	25.50	22.49	19	19.95	20.14
GROC Function	–	–	–	16	7.19	1.05	15	8.27	1.44	19	7.42	2.01	18	7.78	2.16	19	8.32	1.67
GROC Pain	–	–	–	16	6.87	1.26	15	8.00	1.65	19	7.21	2.07	18	7.61	2.09	19	7.89	1.91
Satisfaction	–	–	–	16	10.25	1.29	15	10.33	0.76	19	9.84	1.97	18	9.56	2.50	19	9.84	1.74
6MWT (m)																		
Pre	20	356.88	100.58	16	421.61	82.18	15	450.61	72.17	–	–	–	–	–	–	–	–	–
Post	20	360.20	97.29	16	417.77	80.83	15	465.18	82.50	–	–	–	–	–	–	–	–	–
Pressure pain threshold–mean of eight peripatellar sites (*N*)																		
Most painful knee	20	49.18	34.00	–	–	–	15	59.03	27.19	–	–	–	–	–	–	–	–	–
Least painful knee	20	52.40	34.94	–	–	–	15	64.76	47.72	–	–	–	–	–	–	–	–	–
Pressure pain threshold – standard site over tibialis anterior (*N*)																		
Most painful knee	20	42.68	28.68	–	–	–	15	47.37	25.02	–	–	–	–	–	–	–	–	–
Least painful knee	20	43.73	27.08	–	–	–	15	56.27	23.44	–	–	–	–	–	–	–	–	–

*SPA* sensitivity to physical activity, *WOMAC* Western Ontario and McMaster Universities Osteoarthritis Index, *6MWT* 6-minute walk test, *GROC* global rating of change, *N* Newtons

The results for the piloted primary outcomes for the active phototherapy intervention group are presented for the active phototherapy group in Table 3. The results for the piloted primary outcomes for the placebo control group are presented for the placebo control group in Table 4.

**Table 3:** Outcome measure data from active phototherapy intervention group at all assessment time-points

Measures	Baseline (week 0)			Visit 4 (week2)			Visit 8 (week 4)			4-week follow-up (week 8)			8-week follow-up (week 12)			12-week follow-up (week 16)		
	*N*	Mean	SD	*N*	Mean	SD	*N*	Mean	SD	*N*	Mean	SD	*N*	Mean	SD	*N*	Mean	SD
SPA (peak pain–baseline pain)																		
Pre	10	2.00	1.41	8	0.75	0.89	8	0.50	1.07	–	–	–	–	–	–	–	–	–
Post	10	1.30	1.34	8	0.50	0.93	8	0.38	1.06	–	–	–	–	–	–	–	–	–
Pain severity																		
Worst	10	4.20	2.04	8	2.88	2.42	8	3.00	1.77	9	1.67	1.50	8	1.75	2.32	9	2.44	3.09
At least	10	1.70	1.49	8	0.25	0.46	8	0.25	0.46	9	0.22	0.44	8	0.25	0.46	9	0.44	1.01
Average	10	3.30	1.42	8	1.38	1.06	8	1.13	0.84	9	1.33	1.12	8	0.88	0.99	9	1.22	1.72
Right now	10	3.10	2.13	8	2.13	2.59	8	0.38	0.52	9	0.11	0.33	8	0.75	1.75	9	0.44	3.28
Index	10	3.08	1.95	8	1.66	2.03	8	1.88	1.49	9	0.83	1.16	8	0.91	1.57	9	1.39	2.46
WOMAC																		
Pain	10	5.50	2.12	8	3.25	2.25	8	3.13	2.10	9	2.44	1.42	8	2.38	1.69	9	2.78	2.73
Stiffness	10	3.20	1.75	8	1.50	1.20	8	1.38	1.06	9	2.11	1.27	8	1.62	1.51	9	0.78	0.83
Physical Function	10	20.70	11.82	8	10.25	7.36	8	7.75	5.95	9	11.00	9.15	8	9.63	9.61	9	7.56	7.47
Total	10	29.40	14.29	8	15.00	10.23	8	12.25	8.01	9	15.56	10.85	8	13.63	11.43	9	11.11	10.42
GROC Function	–	–	–	8	7.38	1.30	8	8.63	1.85	9	8.33	1.32	8	8.87	1.25	9	9.11	1.45
GROC Pain	–	–	–	8	7.25	1.39	8	8.25	1.83	9	8.22	1.39	8	8.63	1.30	9	8.67	1.66
Satisfaction	–	–	–	8	10.88	0.35	8	11.00	0.00	9	10.22	1.99	8	10.38	1.41	9	10.44	1.33
6MWT (m)																		
Pre	10	391.78	103.98	8	449.85	63.53	8	469.57	50.71	–	–	–	–	–	–	–	–	–
Post	10	402.62	88.99	8	456.09	64.78	8	489.27	62.21	–	–	–	–	–	–	–	–	–
Pressure pain threshold–mean of periopaterllar sites (*N*)																		
Most painful knee	10	46.49	31.46	–	–	–	8	57.68	29.82	–	–	–	–	–	–	–	–	–
Least painful knee	10	52.09	40.40	–	–	–	8	56.70	27.91	–	–	–	–	–	–	–	–	–
Pressure pain threshold–standard site over tibialis anterior (*N*)																		
Most painful knee	10	47.7	35.83	–	–	–	8	53.38	25.52	–	–	–	–	–	–	–	–	–
Least painful knnee	10	47.25	34.92	–	–	–	8	49.56	26.77	–	–	–	–	–	–	–	–	–

*SPA* sensitivity to physical activity, *WOMAC* Western Ontario and McMaster Universities Osteoarthritis Index, *6MWT* 6-minute walk test, *GROC* global rating of change, *N* Newtons

**Table 4:** Outcome measure data from placebo control group at all assessment time-points

Measures	Baseline (week 0)			Visit 4 (week2)			Visit 8 (week 4)			4-week follow-up (week 8)			8-week follow-up (week 12)			12-week follow-up (week 16)		
	*N*	Mean	SD	*N*	Mean	SD	*N*	Mean	SD	*N*	Mean	SD	*N*	Mean	SD	*N*	Mean	SD
SPA (peak pain–baseline pain)																		
Pre	10	2.35	0.94	8	0.50	0.76	7	0.00	0.16	–	–	–	–	–	–	–	–	–
Post	10	1.35	1.00	8	0.63	0.74	7	0.57	1.27	–	–	–	–	–	–	–	–	–
Pain severity																		
Worst	10	5.30	2.21	8	3.87	2.17	7	3.00	2.00	10	4.40	2.99	10	4.30	2.67	10	3.70	2.41
At least	10	1.70	1.83	8	1.13	1.36	7	0.14	0.38	10	2.40	3.06	10	2.30	2.98	10	1.80	3.05
Average	10	3.80	1.62	8	2.25	1.75	7	1.29	1.25	10	3.40	2.80	10	3.20	2.66	10	2.70	2.67
Right now	10	2.80	1.99	8	1.50	1.07	7	1.57	2.23	10	3.00	3.37	10	2.70	3.37	10	2.10	2.89
Index	10	3.4	2.28	8	2.19	1.89	7	1.50	1.86	10	3.30	3.03	10	3.1	2.92	10	2.6	2.75
WOMAC																		
Pain	10	7.10	2.38	8	5.38	2.26	7	4.14	2.67	10	6.50	4.25	10	7.10	4.33	10	6.10	4.48
Stiffness	10	3.00	0.94	8	3.00	1.51	7	2.00	1.16	10	3.60	1.65	10	3.50	1.96	10	2.70	1.89
Physical Function	10	25.20	6.96	8	21.38	10.03	7	10.71	7.85	10	23.00	20.13	10	24.40	19.12	10	19.10	17.84
Total	10	35.30	8.98	8	29.75	13.01	7	16.86	10.72	10	33.10	25.00	10	35.00	25.07	10	27.90	23.81
GROC Function	–	–	–	8	7.00	0.76	7	7.86	0.69	10	6.60	2.22	10	6.90	2.38	10	7.60	1.58
GROC Pain	–	–	–	8	6.50	1.07	7	7.71	1.50	10	6.30	2.14	10	6.80	2.30	10	7.20	1.93
Satisfaction	–	–	–	8	9.62	1.60	7	9.57	0.98	10	9.50	2.01	10	8.90	3.04	10	9.30	1.95
6MWT (m)																		
Pre	10	321.97	88.53	8	393.37	92.82	7	428.95	90.15	–	–	–	–	–	–	–	–	–
Post	10	317.77	89.80	8	379.44	80.30	6	433.05	100.56	–	–	–	–	–	–	–	–	–
Pressure pain threshold–mean of peripatellar sites (*N*)																		
Most tender knee	10	51.89	36.27	–	–	–	7	60.29	25.01	–	–	–	–	–	–	–	–	–
Least tender knee	10	52.70	28.57	–	–	–	7	73.98	62.05	–	–	–	–	–	–	–	–	–
Pressure pain threshold – standard site over tibialis anterior (*N*)																		
Most tender knee	10	38.10	18.87	–	–	–	7	42.64	12.52	–	–	–	–	–	–	–	–	–
Least tender knee	10	40.20	16.10	–	–	–	7	48.50	18.93	–	–	–	–	–	–	–	–	–

*SPA* sensitivity to physical activity, *WOMAC* Western Ontario and McMaster Universities Osteoarthritis Index, *6MWT* 6-minute walk test, *GROC* global rating of change, *N* Newtons

The results for adherence to prescribed exercise are presented in Table 5. The mean overall adherence to prescribed exercise over the 4-week treatment period was 93.60% (SD 9.36) across all visits among both treatment groups.

**Table 5:** Self-reported adherence to prescribed exercise program

Component of exercise intervention	Aggregated, *n* = 15 (% of prescribed exercises completed over 4-week treatment period)		Active phototherapy, *n* = 8 (% of prescribed exercises completed over 4-week treatment period)		Placebo, *n* = 7 (% of prescribed exercises completed over 4-week treatment period)	
	Mean	SD	Mean	SD	Mean	SD
Chair squats	95.44	6.85	94.34	5.07	96.70	8.72
Step-ups	94.91	5.95	94.34	5.07	95.56	7.20
Lunge	94.38	6.85	93.82	5.57	95.01	8.51
Resisted knee flexion	94.91	6.61	94.34	5.07	95.56	8.43
Single leg stance	95.68	5.02	94.34	5.07	97.21	4.88
Knee flexion/extension range of motion	95.94	4.71	94.34	5.07	97.76	3.83
Walking program	83.97	17.67	91.36	6.59	75.53	22.84
Overall	93.60	9.36	93.84	5.19	93.33	12.60

The results for current pain intensity and adverse events immediately before and after treatment are presented in Table 6. Pre-post differences in pain intensity from before to after the phototherapy for aggregated, active phototherapy, and placebo control groups are presented in Table 7. There were no adverse events reported in either group.

**Table 6:** Immediate effects on pain intensity and adverse effects for aggregated, active phototherapy, and placebo control groups

Visit number^a^		Currnet pain intensity (0 to 10)									Adverse effects—number of participants reporting “yes” to the question “did you experience any adverse effects or side effects during the treatment today?”	
		Aggregated			Active phototherapy			Placebo control			Active Phototherapy	Placebo control
		*N*	Mean	SD	*N*	Mean	SD	*N*	Mean	SD		
Visit 2	Pre-phototherapy	19	1.68	2.11	9	1.33	1.23	10	2.00	2.71	0	0
	Post-phototherapy	19	0.84	1.30	9	0.78	1.09	10	0.90	1.52	0	0
	Post-phototherapy and exercise	19	1.68	1.53	9	1.56	1.33	10	1.80	1.75	0	0
Visit 3	Pre-phototherapy	18	2.06	2.53	9	1.78	2.54	9	2.33	2.65	0	0
	Post-phototherapy	18	1.22	2.05	9	1.11	2.26	9	1.33	1.94	0	0
	Post-phototherapy and exercise	18	1.50	1.58	9	1.22	1.79	9	1.78	1.39	0	0
Visit 5	Pre-phototherapy	15	1.20	1.37	8	1.25	1.28	7	0.43	0.79	0	0
	Post-phototherapy	15	0.40	0.74	8	0.38	0.52	7	0.00	0.00	0	0
	Post-phototherapy and exercise	15	1.07	1.22	8	0.50	1.07	7	0.86	1.22	0	0
Visit 6	Pre-phototherapy	15	0.53	0.64	8	0.38	0.52	7	0.71	0.76	0	0
	Post-phototherapy	15	0.33	0.82	8	0.50	1.07	7	0.14	0.38	0	0
	Post-phototherapy and exercise	15	0.53	0.92	8	0.50	0.76	7	0.57	1.13	0	0
Visit 7	Pre-phototherapy	15	0.47	0.64	8	0.25	0.46	7	0.71	0.76	0	0
	Post-phototherapy	15	0.13	0.35	8	0.13	0.35	7	0.14	0.38	0	0
	Post-phototherapy and exercise	15	0.87	1.25	8	0.38	0.52	7	1.43	1.62	0	0

^a^Visits 1, 4, and 8 did not include pre-post measures for pain intensity. On visits 1, 4, and 8, pain intensity was measused prior to the sensitivity to physical activity (SPA) test which resulted in an increase in pain intensity immediately prior to phototherapy for most participants. Thus, the results of visits 1, 4, and 8 could not be pooled with the results from the other visits

**Table 7:** Change in pain intensity pre-post phototherapy for aggregated, active phototherapy, and placebo control groups

Visit number^a^	Change in pain intensity pre-post phototherapy (0 to 10)								
	Aggregated			Active phototherapy			Placebo control		
	*N*	Mean	SD	*N*	Mean	SD	*N*	Mean	SD
Visit 2	19	− 0.84	1.17	9	− 0.56	0.88	10	− 1.10	1.37
Visit 3	18	− 0.83	1.34	9	− 0.67	1.32	9	− 1.00	1.41
Visit 5	15	− 0.80	1.37	8	− 1.13	1.73	7	− 0.43	0.79
Visit 6	15	− 0.20	1.08	8	0.13	1.25	7	− 0.57	0.79
Visit 7	15	− 0.33	0.49	8	− 0.13	0.35	7	− 0.57	0.53
Mean of means for Visits 2, 3, 5, 6, and 7	–	− 0.60	0.31	–	− 0.47	0.49	–	− 0.73	0.30

^a^Visits 1, 4, and 8 did not include pre-post measures for pain intensity. On visits 1, 4, and 8, pain intensity was measused prior to the sensitivity to physical activity (SPA) test which resulted in an increase in pain intensity immediately prior to phototherapy for most participants. Thus, the results of visits 1, 4, and 8 could not be pooled with the results from the other visits

## Discussion

Our results suggest that a future randomized controlled trial evaluating the effectiveness of active phototherapy intervention versus placebo control in a population with knee OA is feasible. We achieved a recruitment rate of 6.7 participants/week over 3 weeks from two primary care sites. At this rate, a fully powered randomized controlled trial with 168 participants could be recruited in approximately 25 weeks, which would provide 90% power to detect a moderate effect size difference between groups with an alpha of 0.05. The retention rate of 95% at the end of study and overall assessment completion rate of 89% also exceeded our a priori target for proceeding with the full trial. Finally, the pilot study demonstrated strong treatment fidelity with 100% of treatment components delivered in alignment with the study protocol across all completed visits.

One challenge experienced in this trial was lower assessment completion rates for in-person assessments at 2 and 4 weeks (80% and 75% completion respectively) in comparison to online follow-up assessments in which 90% or greater completion rates were maintained. This is important, especially given that the primary outcome of the future RCT is a measure of pain during a standardized 6MWT, which requires in person testing. The challenges in attending in person assessments was largely due to the medical complexity of the sample participating in the study. One person dropped out of the study prior to the 2-week assessment, reporting a severe illness that was not directly related to their OA. The other four participants who did not complete the 4-week assessment all missed their appointments due to comorbid health conditions affecting them or their partner. The high completion rate of online assessments despite these ongoing challenges for participants was an indicator of commitment of participants to participating in the study. Therefore, we feel confident that the information and consent process effectively informed participants and that changing the information and consent process would not improve the in-person completion rate. We also discussed how changes to the inclusion or exclusion criteria may have helped to identify participants with multiple comorbidities more likely to experience health complications that could interfere with participation. However, all investigators agreed that the strength of this study was the recruitment of participants through primary care settings to best represent the population of people with OA seeking care, who often experience multiple comorbidities [65–70].

The finding of lower rates of assessment completion at in-person visits will inform the fully powered trial in three ways. (1) We will aim to recruit from primary care sites where they have the physical space available to complete the 6MWT onsite. By offering participants with barriers to attending in person at the university lab the opportunity to complete the assessment in conjunction with another healthcare appointment at their primary care site, we may be able to help participants overcome geographic barriers to participation which may increase our rate of competition of the primary outcome. This is a strategy the research team has implemented successfully in other trials. (2) We will follow-up multiple times via email and telephone with participants who are unable to attend in-person assessments to ensure that we still achieve high rates of completion of self-report measures at those time-points, even if we are not able to capture the performance measures. 3) We will update our sample size calculation in order to allow for up to 25% dropout. These strategies will be implemented for a fully powered trial to address the challenges of achieving high assessment completion rate for the in-person assessments, while maintaining the strength of the study in representing the population of people with OA who are seeking care to facilitate generalizability of the results.

This study has important limitations that must be acknowledged. First, although our primary outcome piloted for a future trial (SPA) has demonstrated to be cross-sectionally related to disability in adults with knee OA [50], the SPA measure we used measures change in pain during a walking task only. Change in pain during walking may not fully capture the complexity of pain during physical activity reported by people living with knee OA. Second, we did not control for co-intervention (e.g., intra-articular joint injections) among participants in this research. As such, it is possible that co-interventions may have been a confounding factor which impacted the results. We plan to use a pragmatic design, but will plan a sensitivity analysis for the full trial to assess whether the effect of the intervention is influenced by co-interventions. Third, our inclusion criteria indicated that participants had to receive a diagnosis of knee OA from a physician. We did not specify that participants needed to be radiographically diagnosed in order to meet inclusion criteria. As such, it is possible that some participants in this research could have had knee pain diagnosed as knee OA, but without radiographic evidence of knee OA. Our fully powered trial will include radiographic evidence within the inclusion criteria.

## Conclusion

The results of this pilot study suggest that it is feasible to implement a fully powered randomized controlled trial to evaluate the effectiveness of an active phototherapy intervention versus placebo at reducing pain during movement in a population with knee OA.

## Data Availability

The datasets used and/or analyzed in this research are available from the corresponding author upon request.

## Abbreviations

RCT: Randomized controlled trial
OA: Osteoarthritis
TENS: Transcutaneous electrical nerve stimulation
mW/cm^2^: Milliwatt per square centimeter
SPA: Sensitivity to physical activity
6MWT: Six-minute walk test
NPRS: Numeric pain rating scale
BPI: Brief Pain Inventory
WOMAC: Western Ontario and McMaster Universities Ostoearthritis Index
GROC: Global rating of change
N: Newtons
SD: Standard deviation
NSAID: Non-steroidal anti-inflammatory drug
IQR: Interquartile range
